# Considering Fraud Vulnerability Associated with Credence-Based Products Such as Organic Food

**DOI:** 10.3390/foods10081879

**Published:** 2021-08-14

**Authors:** Louise Manning, Aleksandra Kowalska

**Affiliations:** 1 School of Agriculture, Food and the Environment, Royal Agricultural University, Stroud Road, Cirencester, Gloucestershire GL7 6JS, UK; 2 Institute of Economics and Finance, Maria Curie-Skłodowska University, pl. Marii Curie-Skłodowskiej 5, 20-031 Lublin, Poland; aleksandra.kowalska@umcs.lublin.pl

**Keywords:** fraud, vulnerability, credence-based, organic, substitution, organized crime group

## Abstract

Organic foods carry a premium price. They are credence-based foods, i.e., it is difficult for consumers to evaluate the premium aspects of organic food under normal use. In global supply chains, organic food is purchased on institutional trust (certification, logos, standards) rather than on relational trust. Relying on institutional trust makes consumers vulnerable to criminals who intentionally label conventional product as organic or develop sophisticated organized crime networks to defraud businesses and consumers. The aim of this research is to explore cases of organic fraud that are emergent from academic and gray literature searches to identify ways to strengthen future capabilities to counter illicit activities in a globalized food environment. Each case is considered in terms of perpetrator motivations (differentiated as economic, cultural, and behaviorally orientated drivers), the mode of operation (simple or organized), the guardians involved/absent, and the business and supply chain level vulnerabilities the cases highlight. The study finds that institutional trust is particularly vulnerable to fraud. Supply chain guardians need to recognize this vulnerability and implement effective controls to reduce the likelihood of occurrence. However, in some cases considered in the study, the guardians themselves were complicit in the illicit behavior, further increasing consumer vulnerability. Future research needs to consider how additional controls can be implemented, without increasing supply chain friction that will impact on food trade and supply, that can ensure consumers are purchasing what they believe they are paying for.

## 1. Introduction

Organic food production is a method of sustainable food production, often legally defined, which contributes to environmental and animal welfare protection via a set of specific agricultural practices. Consumer concerns about animal welfare [1,2,3,4]; the use of pesticides [2,3,5,6], genetically modified organisms [7]; fertilizers [6]; hormones [5,7]; antibiotics and other veterinary drug use [5,7], and their potential impact on personal and family health [8,9,10,11,12,13] are important antecedents that positively drive the purchase and consumption of organic foods. Consumers who are aware of sustainability issues, including the need to conserve soil, care for the rural countryside, and promote local food supply [5] are also more willing to buy environmentally friendly and ethical products [14] such as organic foods.

Organic foods usually carry a premium price compared with their conventional equivalents [15,16]. Organic farming has higher benefit/cost ratios (20–24%) than conventional agriculture [17], however, the difference in the farm-input costs between organic and conventional agriculture depends on the crop grown [17,18]. This price differential is often rationalized in food markets as organic farm production having lower yields than conventional production, higher production costs and increased certification costs [5,19]. Private organizations (acting privately or on behalf of regulators) or inspectors that have regulatory oversight for governments at national or local level [15] inspect organic producers on a regular basis. However, the premium price for organic food, as with any credence food, creates a vulnerability for fraud [20]. Consumers cannot assess credence characteristics in the purchasing, preparation, or consumption of food, i.e., they rely on trust. Credence characteristics might include “whether livestock have access to grazing, and if so for how many days per year; or how much space an animal has been afforded in a given environment” [20] (p. 68). Verifying of credence characteristics can be relational (I know the person) or institutional via certification, or regulatory standards [21,22,23]. 

Inherent failures in credence goods markets are of interest in the literature regarding the quality of product or service certification [24,25]; especially with organic foods [26]. Certification is communicated to business via documentation and certificates and to consumers in the form of logos, cues or other information on packaging [26]. Consumers need to trust supply chain actors and regulators that institutional approaches are in place, are validated and verified, and that food businesses are not undertaking any illegal activity to mislead or cheat customers. Whilst institutional trust can develop consumer confidence in regulatory, inspections and market systems of enforcement, and surveillance; it provides confidence by default, primarily via the absence of incidents. Institutional trust is also promoted through generalized symbols, brands, official control standards [27], labeling and cues [28,29], it lacks the “face-to-face validity” of relational trust and is vulnerable, without suitable controls, to disruption and fraud [29,30].

The aim of this research is to explore cases of organic food related fraud that are emergent from academic and gray literature searches to identify ways to strengthen future capabilities to counter illicit activities in a globalized food environment. Each case is considered in terms of perpetrator motivations (differentiated as economic, social, and behaviorally orientated drivers), the guardians involved/absent (regulatory and enforcement and supply chain surveillance), the model of operation (simple model or an example of the activities of an organized criminal group or OCG) and the business and supply chain level vulnerabilities the cases highlight. A case study-based narrative is developed and key perpetrator motivations for committing fraud associated with organic food products are differentiated as economic, cultural, and behaviorally orientated drivers [15].

## 2. Literature Review

Organic food production is defined within regulations in many countries such as the United States of America (US) or trading areas such as the European Union (EU). Private market standards also define organic production standards, and private organic certification processes need to comply with regulatory requirements. In the EU, Regulation (EC) No. 834/2007 [31] and the corresponding implementing regulations define the principles of organic production, certification, and labeling with an associated mandatory logo, see Regulation (EC) No. 271/2010 [32], as subsequently amended. Regulation (EU) 2018/848 on organic production and labeling of organic products [33] repeals Regulation (EC) 834/2007 and was to be applied from 1 January 2021. However, the date for adoption is deferred by one year because of the COVID-19 pandemic (Regulation (EU) 2020/1693 [34]). While organic food products sold in the EU must comply with EU legislation, private organizations are able to determine their own organic standards that exceed regulatory requirements [32] and can request additional compliance criteria in terms of how food is produced and particularly how animals within food production live their lives. 

There are no common regulatory or private agreements on organic food production standards. The need for clear and harmonized rules and principles has not only been taken up by private bodies, such as IFOAM—Organics International, and state authorities, but also by United Nations organizations, including the Food and Agriculture Organization (FAO), the World Health Organization (WHO), and the United Nations Conference on Trade and Development (UNCTAD) [35,36]. Despite this, harmonization can occur through signing bilateral equivalency trade agreements between countries with agreed organic food standards. The EU has signed equivalency trade agreements for organic products (so another country’s control system and its standards are in line with domestic requirements and the products certified in those countries can be sold on the national market) with Canada, US, and Switzerland, Argentina, Australia, Costa Rica, India, Israel, Japan, Switzerland, Tunisia, and New Zealand [35,36,37]. However, the US and the EU have not recognized each other’s national organic standards and control systems for animal products from the EU, apples and pears from the US, wine, and aquaculture products, hence this approach could be described as a “restricted partial equivalency” [36,37]. In 2019, 17 countries were still in the process of drafting organic food legislation, including Bosna and Herzegovina, Egypt, Bangladesh, Jordan, Nepal, Pakistan, and Sri Lanka. 

Awareness of the lack of consensus in organic labeling could influence consumer trust in standards. Indeed, previous research has highlighted levels of consumer distrust of certification bodies and labeling of organic food [2,3,38,39]. Trust as a construct reduces complexity in consumer decision-making. Misplaced consumer trust can lead to economic harm, harm to the brand if the incident is identified in the media [40], and personal harm in the event of a food safety related incident [41,42]. There are greater levels of consumer trust for some organic logos more than others [32]; and differential trust levels depend on the degree of sophistication of the organic market in the country concerned [43]. For example, in China, organic certification was seen as trustworthy, but more so by women and with a variance in level of trust by age, education and city location [44]. In researching consumer distrust associated with food, Giampietri et al. [41] (p. 161) consider the dissociation between consumers and farmers and state:

“Food safety and quality currently represent a black box for consumers, especially for those who live in urban areas that, by their very nature, are quite far from the production process and who have gradually lost their control over food”.

Organic food is therefore a suitable lens of enquiry to consider credence-based foods that experience ex post information asymmetries [20,32], and are vulnerable to fraud. The potential for fraud in the organic food supply chain is highlighted in multiple academic and industry sources [15,45,46,47,48]. In the US, a range of both domestic and international ingredients and foods have been identified by the United States Department of Agriculture (USDA) as being fraudulently labeled or certified as “organic” [46]. Economically motivated adulteration (EMA), a subcategory of food fraud, is intentional deception associated with food products, ingredients, and packaging for economic gain, that includes activities such as substitution especially with substandard or inferior products, unapproved additions or enhancements, misbranding or misrepresentation, tampering, counterfeiting, using stolen goods, and others [47,49,50,51,52]. The UK National Food Crime Unit [53] identify seven types of food crime: theft, illegal processing, waste diversion, adulteration, substitution, misrepresentation (marketing or labelling a product to wrongly portray its quality, safety, origin, or freshness), and document fraud (making, using, or possessing false documents with the intent to sell or market a fraudulent or substandard product). These crimes can be isolated, informal or can be activities associated with highly organized networks. An OCG is defined by the United Nations Convention against Transnational Organized Crime [54] as being “a structured group of three or more persons, existing for a period of time and acting in concert with the aim of committing one or more serious crimes or offences… in order to obtain, directly or indirectly, a financial or other material benefit”. OCGs vary in their physical structure and mode of operation i.e., types of illicit activities and enterprise, operating in country, or across multiple borders, through historical, cultural (values and language), kinship, and ethnic connections [55,56,57,58]. 

So far, there is no harmonized definition of food fraud at the EU and international level which makes it challenging to comprehend, communicate, and ideate on how to prevent it [59]. Food fraud definitions collectively share common themes of intentional acts of deception, as aforementioned, and more widely smuggling, gray market/diversion, and counterfeiting (intellectual property rights) [49,59,60,61,62]. These differences in private and regulatory approaches to food crime classification pose limitations in the comparative analysis of food fraud data in different jurisdictions, but this should not understate the importance of these recognized initiatives, which evolve from the joint activities of governments and the private sector. 

*Motivation* to commit fraud with organic foods is associated with the degree and nature of information asymmetry in the supplier–customer (perpetrator-victim) relationship. This relationship especially the degree to which the perpetrator expects/intends for there to be return or repeat sales, market arrangements and competing interests will frame the potential for fraudulent activity [20] in organic food supply chains. Motivating factors that influence food fraud (Table 1) are synthesized from the literature reviewed in this study and are characterized as economic drivers and cultural and behavioral drivers [15].

*Vulnerability* is the extent to which an individual, organization, supply chain or national food system is at risk from, or susceptible to, attack, emotional injury or physical harm, or damage from intentional illicit activity [63]. Much has been written about the vulnerability of organic foods to instances of fraud [15], and this can be extended to other credence-based foods such as kosher and halal [64], mediated by the characteristics of individual sectors and the organizations themselves [65]. Product testing protocols are being developed to identify conventional food posing as its organic counterpart but introducing product testing can increase costs and friction in global supply chains and reduce the degree of acceptance of institutional trust mechanisms. *Guardians* monitor and protect food, processes, organizations, supply chains, and nations against illicit activity [66] and supply chain guardians need to recognize the economic, cultural, and behavioral drivers of food fraud and implement effective controls to reduce the likelihood of its occurrence. There is limited research on the mechanisms to create greater consumer awareness of the potential for food fraud [67] and ways to recognize and to reduce OCG activity with organic food supply chains. 

Food companies are vulnerable to third parties, such as a supplier delivering inferior, mislabeled, substituted, or adulterated foods or food ingredients [68,69]. Widely known recommendations to tackle food fraud through food safety management systems require organizations to have a documented food crime/fraud vulnerability assessment procedure in place (built on risk management methodology) [70]. Food companies use their own in-house food crime/fraud vulnerability assessment tools or methodologies provided by the recognized organizations or experts such as Threat Assessment and Critical Control Points (TACCP), Vulnerability Assessment and Critical Control Points (VACCP), Safe Supply of Affordable Food Everywhere (SSAFE), and the Food Fraud Initial Screening Model (FFISM) [69,71,72,73,74]. The first two methodologies align with Hazard Analysis Critical Control Points (HACCP), a globally accepted and effective approach against accidental food contamination, making adoption of TACCP/VACCP guidelines easier for an organization. In contrast to HACCP, TACCP focuses particularly on people (e.g., suppliers, employees) [69]. The adoption of TACCP and VACCP is voluntary in many countries, whereas all food facilities in the US must establish and implement an adequate HARPC (Hazard Analysis and Risk-Based Preventive Controls) plan, which means that they have to identify food safety and adulteration hazards, implement controls, design and implement corrective actions, and verify the plan. Crime vulnerability in this context is defined as “the extent to which an individual, organization, supply chain or national food system is at risk from, or susceptible to, attack, emotional injury or physical harm, or damage from an intentional act” [72] (p. 825).

In summary, fraud associated with organic food firstly deceives consumers who are then in reality paying for premium products they do not get in practice. Secondly such illicit practices economically harm organic farmers who are producing to organic production standards, often at higher costs and then facing unfair competition with other actors who are committing fraud and are accessing the market with product that is wholly or in part misleading. Fraudulent products are produced to lower standards with a lower cost structure [15]. Food fraud scandals may even result in substantial damage to the reputation of the entire organic food industry [75]. Thirdly such poor practices undermine confidence in food products certified as being organic ultimately eroding trust and reducing the value associated with the credence characteristics of the product [15]. So, what can we learn from past cases of organic food related fraud where data are available in the public domain?

## 3. Materials and Methods

To serve the aim of this article, a case study-based narrative has been developed. Case study analysis is an accepted method for considering business fraud [56,57,58] and is frequently used as a qualitative research method. An explanatory case study consists of the following steps: (a) a clear account of the facts associated with the case, (b) reflection on the alternative explanations of the facts, and (c) the formation of a conclusion based on the most appropriate explanation of the findings [76]. In itself, a case study can provide both qualitative and quantitative evidence of a particular phenomenon [77]. A business case study is purely suggestive of what might be representative of a wider cross-section of businesses so findings cannot be generalized, but they can provide the basis for new thinking and theory [78]. Whilst Yin [76,77] follows an implicit positivist approach to case studies, Ragin, [79], describes the approach as more emergent, interpretivist and a process of casing i.e., illustrative rather than looking at fixed, bounded cases specific to a given time and place [80]. In this instance of fraud in organic food supply chains, it requires analysis of what ‘the cases are a case of’ [81]. Alternatively, ‘casing’ has been described as systematic combining [82], to allow for problematization [81] and is the approach followed in this study allowing for a more holistic enquiry that seeks to be exploratory, and explanatory [83]. Other studies have used intelligence derived from on-line media sources [84,85] to provide insight into existing phenomenon informing causal investigation [65]. The five cases highlight common and differentiated case characteristics and how these characteristics frame the incidents, and the wider positioning of how organic food fraud may occur [75].

Search terms such as credence AND/OR food AND/OR organic AND/OR consumer AND/OR certification AND/OR trust were used to create a snowball, iterative academic literature review [84]. The iterative search for gray and trade information on organic food fraud in Google continued using the range of search terms until data saturation was reached i.e., no further ‘real-life’ cases were evident in the Google searches. This literature combined with contemporary food fraud literature formed the theoretical grounding of the study. The second stage of the research was to identify case study examples of incidents associated with organic food from 2003 to 2019. Incidents identified in the gray and academic literature were screened on the basis of whether there was sufficient publicly available evidence to determine perpetrator motivations (economic, cultural, and behavioral); the scope of the activity and whether it was national or global, the mode of operation (simple or an example of an OCG network); who the guardians were and then if the vulnerability could be determined. This screening led to the five cases compared in Table 2. In cases of fraud, it is important to note it can be an intentional organizational strategy or an intentional strategy that works at the network level. The five cases are not designed to be an exhaustive list of incidents, but to inform a casing process [81]. Wolf and Hermanson’s four-element fraud diamond model is used to position the five cases in this study [86]. The four elements are pressure, capability, opportunity, and motivation. The case evidence being drawn mainly from the gray literature has an associated limitation that only publicly available information could be considered. Another limitation of this approach is the risk of selection bias, and that the search was only undertaken in the English language, so vital cases could have been lost, and this is considered in the analysis of the results. Deterrence is the inhibition of fraud through the application of appropriate measures that prevent or discourage the fraudulent activity [87].

## 4. Results

This section of the paper considers examples of food fraud associated with organic food. Some of the motivating factors (Table 1 and Table 2) are common across all cases. These include economic drivers such as the price asymmetry between organic and conventional food products creating economic pressure and the motivation to substitute, mislead or deceive; and cultural and behavioral drivers such as the ethical culture of the organization. For fraud to be effectively executed, in each case there must be a capability and an opportunity to commit the fraud and for it to remain unnoticed by guardians in the supply chain, such as third-party certification bodies, regulators, and individuals that work in food supply chain.

### 4.1. Organic Feed Incident, US (2011–2017)

Between 2011 and 2017 the largest known US organic food fraud occurred that not only misled consumers, farmers who purchased and paid for organic feed ingredients they believed were organically produced, but also farmers who were playing by the rules [88]. The farmers grew conventional corn and soybeans, and then after harvest this produce was mixed with certified organic grain, diluting the organic grain and falsely marketing the whole consignment as USDA certified organic product [89]. Prosecutors stated the scheme may have involved up to 7 percent of organic corn grown in the US in 2016 and 8 percent of the organic soybeans [89]. The businessman at the center of the fraud, was sentenced to 10 years in prison for organic fraud and ordered to forfeit USD 128 million [90]. In 2007, with a previous incident with a consignment of soybeans stated as being organic, but found later to be genetically modified, the USDA did not take action [90]. This case again highlights the vulnerability associated with an absence of deterrence and the weakness in the mediating role of institutional trust owned by regulation, enforcement, and surveillance systems. This example again shows three elements of the fraud diamond, capability, opportunity, and motivation [86]. For such cases to be successfully enacted, the perpetrators needed to have knowledge of existing activities to verify organic products and how illegal activity could pass unnoticed. This example operates at the national level.

### 4.2. Organic Raspberries Incident, Chile, (2017)

In January 2017, following a tip-off from a whistleblower, Chilean Customs raided the offices of Frutti di Bosco, a fruit trading company in Santiago [91]. Documentation, data, and traceability records were seized evidencing a global operation centered on raspberries. Hundreds of tons of frozen berries grown in China were shipped to a packing plant in central Chile repackaged and rebranded by Frutti di Bosco as premium Chilean-grown organic fruit, then shipped to consumers in Canadian cities including Vancouver and Montreal [91]. Chilean Customs calculated that at least USD 12 million worth of mislabeled raspberries were sent to Canada between 2014 and 2016. Further, documentary evidence suggested that the berries came from Harbin Gaotai Food Co Ltd., a Chinese supplier that was later linked to a 2017 norovirus outbreak in Quebec that sickened 615 people and fifteen cases in Minnesota in 2016 [92,93]. These raspberries were not identified as organic in the press release. Canadian authorities issued a recall on Harbin Gaotai berries coming directly to Canada from China dating back to July 2016. This example shows the three elements of the fraud diamond, capability, opportunity, and motivation [86]. This case shows again the weakness in the mediating role of institutional trust owned by regulation, enforcement, and surveillance systems against intentional deception. There was a clear economic driver for mislabeling an absence of deterrence and an opportunity to hide illegal activity in long and complex cross-border supply chains. The inability at the time to test product to determine organic status meant there was a vulnerability for businesses and consumers in relying on documentation that could be fraudulent.

### 4.3. Organic Egg Incident, UK and Germany (Multiple Dates)

Egg related fraud i.e., selling cage-produced eggs as free range or organic products is a lucrative activity, creating an incentive for the unscrupulous to commit fraud. In 2010, in the UK, a businessman was jailed for three years after admitting mislabeling caged eggs as free range or organic. Around 100 million mislabeled eggs were sold to retailers over 2 years with a derived additional profit of GBP 3 million [94]. Pidd [94] states: 

“The fraud came to light in 2004 when allegations began circulating in the egg industry that there were vastly more British free range and organic eggs being sold in shops than could ever possibly be laid in UK farms. At the same time, investigators from the Egg Marketing Inspectorate noticed during routine checks that eggs coming from Heart of England were not at all what they purported to be.” The key driver for fraud in this example, and others, is the economic value/financial return derived from intentionally mislabeling eggs as organic or free range. In early 2013, German authorities said they had identified more than 200 farms suspected of selling premium priced eggs as organic free range that were laid by hens said not to conform to organic regulations [95]. These unrelated examples again show the vulnerability of institutional trust to fraud. Capability, opportunity, and motivation can all be demonstrated in this example, pressure from supply chain economics is not identifiable from the case evidence collated [86], but the lack of deterrence is clear. In this example too, guardians became aware as a result of supply chain intelligence, which came to their attention. The next examples consider complex cross-border criminal networks.

### 4.4. Organic Pistachios Incident, Spain, (2019)

An operation began in 2019 following supply chain intelligence and reports of ecological certifications being misused on pistachios that did not adhere to prescribed organic agricultural standards [96]. The Spanish Guardia Civil, together with the French Gendarmerie Nationale and Europol found an OCG involved in the production, distribution, and sale of alleged organic pistachios [97]. The Spanish Guardia Civil detected the mixing of organic and conventional pistachio nuts. Pesticide residues including glyphosate and chlorate were identified in the product and these products were being used to improve the quality and quantity of harvests and increase the monetary value of the production. Marketed as organic, the nuts were sold for up to 80% over the retail price of conventional pistachios [97]. The nuts were also sold in France under false organic certifications. The investigation led to 14 arrests in Ciudad Real, Madrid and Malaga (Spain) for fraud against public health, money laundering, falsification of documents and crime related to market and consumers. It is estimated this case created a profit of EUR 6 million [97]. The guardians in this case were across national boundaries: Spanish Guardia Civil, French Gendarmerie Nationale; Europol; (regulator and enforcement), and supply chain actors (surveillance). There was an intention to defraud by various means including product dilution, false declarations, and money laundering. This example highlights capability, opportunity, and motivation [86], and a process of circumventing guardians and measures in the supply chain. 

### 4.5. Puss in Boots, Green War, Vertical Bio Incident, Italy, (2007–2011)

The 2011 “Gatto con gli stivali” or “Puss in Boots” investigation by the Guardia di finanza (Italian Financial Guard) suggested that between 2007 and 2011 around 700,000 tons of Italian and Romanian sourced cereals, soy and other pulses were fraudulently certified as organic [98]. The estimated value of this trade was EUR 200 million and alleged practices included falsification of documentation as well frequent changes of use of organic certification bodies [98]. In addition to business operators, employees of organic certification bodies were also involved in the fraud that encompassed counterfeiting of certificates, counterfeiting certification documents e.g., production plan, land title deed, land lease agreement, counterfeiting of trade documents, e.g., delivery contracts, delivery notes, or invoices and issuing certificates for crops that had not been grown by the farmers [99]. The fraudulent operators established “short term” companies trading organic products and after their intentional closure it was not possible for the certification bodies to reconstruct the trade flow of goods along the whole supply chain [99]. For a wider study on mislabeling of cereals and bakery products see [52].

The Italian Ministry of Agriculture, Central Inspectorate for the protection of food quality and prevention of fraud (ICQRF) in cooperation with the Italian Financial Guard enacted the Green War inquiry reporting in April 2013 [98,100]. Multiple organizations were involved, and the fraud was undertaken in a complex and highly organized way [77]. The organic certification fraud focused on cereals and oil grains for both food and feed including soy (from India and Moldova), corn (from Ukraine), soft wheat, and flax reported to be genetically modified and to contain unauthorized substances [98,101] but being sold as organic. Products were imported from Third Countries (Moldova, Ukraine, and India) via an Italian company based in Malta [98,101]. A further investigative operation Vertical Bio in 2014 highlighted imported certified cereals and oil grains from Moldova, Ukraine, Kazakhstan and India that were potentially fraudulent [98]. Police investigations concluded that two OCGs were involved in the import and sale of products stated to be “organic” in Italy, via a Maltese registered export company [101]. Key to the success of this illicit network was the “free market” aspects of the regulatory controls in the EU. This meant that products approved in Malta could then be sold across the EU without further inspection. Between 2007 and 2013, the companies involved imported some 350,000 tons of corn, soybeans, wheat, rapeseed, and sunflower seeds, at an estimated market value of EUR 126 million [101]. The degree of deceit included false documentation and certification of conventional product being sold as organic via employees of certification bodies; an organized activity that went beyond substitution to the interchanging of trading companies and developing documentation for farms that did not exist. 

This case highlights OCGs operating through supply networks where institutional trust guardians such as third-party certification companies were set up to imply governance oversight and were indeed actors that were a key part of the mode of execution of the crime. Another recent example, albeit not organic food, where regulatory guardians were involved in the crime itself is the “weak meat” scandal associated with JBS in Brazil [102]. The 2-year investigation covered six Brazilian states and Brasília and 33 officials were suspended from the Brazilian Ministry of Agriculture [102].

### 4.6. Summary 

These case studies highlight the vulnerabilities that arise, the modes of operation and how they are framed by opportunity, capability, and motivation. In 2018, the European Commission (EC) and Europol launched an investigation into organic food supply chain integrity within the framework of the OPSON VIII operation [103]. The investigation (December 2018–April 2019) aimed to identify vulnerable points [87] within the international food and feed organic supply chain with special emphasis on traceability, integrity, and false certification [104]. The outcome of the investigation was that several administrative and criminal proceedings were initiated, products were seized (EUR 100 million 16,000 tons and 33 million liters), people were arrested (672 people) and operators sanctioned [105]. With regard to organic products, more than 90,000 tons were checked and 9 individuals arrested by the Spanish police [105]. There were 12 criminal investigations, two court cases, two financial investigations, and two OCGs were identified and broken up [104]. The new Official Control Regulation combined with new Organic Regulations due to come into force in 2021 will strengthen controls within the EU [103], but what vulnerabilities will remain?

## 5. Discussion

Embedded credence-based attributes add value to organic food products (including healthiness, food safety, environmental protection, animal welfare) but also create increasing fraud vulnerability as shown in all the presented case studies. The five examples show both single organizational examples and complex OCG networks. Next, we make a detailed critical analysis of two exemplary cases, i.e., UK and German egg fraud, and organic raspberries fraud, to demonstrate how to utilize contextual information for in-depth analysis of the cases, and a to provide a detailed diagnosis of the factors which might impact on the integrity of the organic food supply chain.

Considering the organic feed fraud incident in the US, supply volume and pricing of raw material might have been the main perpetrator motivation for deceptive behavior as the scarcity of organic feed in the market and difficulties for the feed industry to become organic were identified as major issues that hinder an increase of global organic livestock production [106]. This growth in the market and the challenges of supply can also be seen in the 2000s with the growth of free range and organic egg sales. There has been a continued growth of free-range eggs production (including organic eggs) in the UK in the 2000s resulting from growing consumer demand and the EU-wide ban on intensive battery housing [107]. Several supermarkets in the UK, as well as other food service and retail corporations, adopted a cage-free egg policy in the late 1990s and the beginning of the 2000s. By 2005, free-range production constituted 30% of egg sales in the UK, and free-range eggs reached 56% of egg sales in 2019 [108]. As consumer demand for organic eggs reached significant proportions of the total retail egg market (about 30% in Denmark and France in 2017, 27% in Switzerland, and only 7% in the UK in 2019), this created a market opportunity for EMA, particularly mislabeling [109]. The ongoing price competition between retailers also led to reduced margins for free-range eggs and price premiums for organic eggs [108]. This economic pressure is a key perpetrator motivation to undertake illicit behavior. Hence, high levels of competition in the sector might have been a key food fraud factor in the UK/German organic egg fraud incident. 

In the case of 2017 organic raspberries fraud, it is important to differentiate between perpetrator and “victim” companies from Chile and Canada i.e., those who were not directly involved in food fraud, but were victimized by their supplier’s activities. The victimized company, especially if they are unaware of the fraud, have no ability to address the factors that motivate the crime within their supply chain. Indeed, many of the motivating factors may be out of their control [71] e.g., in some of the cases discussed herein, the guardians themselves may be complicit in the illicit behavior. The cases included in this paper had a high degree of organization i.e., they were not occasional or random in nature. The perpetrators were also driven by a range of factors: economic (supply and demand dynamics, price and information asymmetry, competitive advantage of illicit behavior, and changes in financial constraints on the supply chain), and cultural and behavioral drivers (ethical, cultural, and accepted business practice) [15]. These examples show the dynamic, entrepreneurial nature of food fraud and the vulnerability legitimate food organizations and consumers face. Institutional trust has been shown to be particularly vulnerable to fraud, especially in long, complex, cross-border supply chains. Regulatory and supply chain guardians need to recognize this vulnerability and implement effective controls to reduce the likelihood of occurrence. These controls must be dynamic as perpetrators of fraud will continually seek to overcome actions taken to limit their activity especially when such operations prove so lucrative.

There are several trust-in-(organic) food arrangements set up in different parts of the world. Zhang et al. [27] distinguished three ideal types of trust regimes: government-based trust regime, market-based trust regime, and civil society-based trust regime which involve government authorities, enterprises, both privately or publicly owned, civil-society organizations and consumers in various combinations. Government-based trust regimes rely on sufficient and effective resources being provided to national and local authorities to prevent food producers from selling low-quality, unsafe, or dangerous food [27]. Effective regulation regarding the organic food supply chain is primarily based on establishing science-based food quality and safety standards and sufficiently strong regulatory control mechanisms to prevent non-compliance. Market-based trust regimes use private certification schemes, shown in this work to be vulnerable to circumvention, and organizational procurement policies and processes that are enacted by organizations across their supply base. Civil society-based trust-in-food regimes may include in particular: awareness campaigns around certification and labelling schemes (e.g., organic), consumer guides, and web-based information schemes. These three aspects need to co-align to prevent organic food fraud incidents and mitigate the adverse effects of such cases on the market for organic food.

EU food law to prevent fraudulent or deceptive practices, the adulteration of food, and any other practices which may mislead the consumer, protects consumers’ interests, so national practices and legal regimes should provide for clear and transparent process of imposing a (financial) penalty due to the detection of mislabeling of food. It is essential to establish an effective system of sanctions to deter perpetrators and to hold them accountable for their actions. The revenue derived by the perpetrator should become a fundamental criterion to take into account when considering the magnitude of the financial penalty. For instance, in Poland the penalty payment will be calculated depending on the degree of harmfulness, the degree of culpability, the scope of infringement, the producer’s activities to date and the production volume of the operator [110]. It is worth adding that that EU food law was designed to deal with food safety incidents rather than food fraud and/or adulteration issues, and the interpretations of Regulation (EC) 178/2002 happen to differ considerably among the Member States where a food crime incident takes place. This, in consequence, results in food recalls in some countries and no further action being taken in respect of the case in other countries [111]. This lack of harmonization promotes an environment where illicit behavior can occur, even flourish.

Another option is to promote food crime/fraud vulnerability assessment tools or methodologies provided by the recognized institutions such as the use of TACCP, VACCP, SSAFE, or FFISM, and to provide support to the participation of potential “food fraud experts” in relevant courses in that field [52,93,112]. This may include government aid to training courses for owner-managers of micro, small and medium-sized enterprises. Consumer education is a further component to mitigating food fraud vulnerabilities, e.g., through knowledge and information-dissemination on existing sources of data on food law non-compliance. The aim behind this is to make the market itself more effective force for preventing food crime/fraud. Developing legal frameworks and market-based mechanisms and providing the appropriate resources for their implementation should serve a stronger protection of consumers (in terms of their health, social and economic interest) and businesses in terms of embedding fair trade practices.

The UK Food Standards Agency through the NFCU has developed an on-line free Food Fraud Resilience Self-Assessment Tool [113] that should be completed by organizations in line with a formal counter-fraud strategy [114] that also includes ensuring that their supply base has a similar strategy and that as a business the zero-tolerance of fraud is communicated e.g., in policies or contracts as well as the sanctions or penalties that would occur in a breach of the policy [113]. This is one example of a regulatory guardian response to support organizations in addressing their vulnerability to food fraud. The vulnerability to food fraud extends beyond credence-based foods to all foods so effective counter-fraud strategies need to be in place. The lack of consensus on developing a standardized process for food fraud vulnerability assessment needs to be addressed [115,116]. The case studies in this paper however show that whilst some organizations can develop counter-fraud strategies to address organic food fraud, by the nature of the fraud undertaken (substitution, mislabeling); the organizations involved will still seek to deceive others especially if there is a reliance on institutional trust.

The OCG examples show that organizational vulnerability for organic food fraud extends beyond developing protocols to minimize vulnerability to purchasing mislabeled, substituted materials as some organizations become involved in highly connected and organized criminal networks. Perpetrators in these criminal activities are professionalized in that the criminal activity is fully funding their lifestyle. Food Crime Risk Assessment has been proposed as a tool that should be developed for food businesses [72] and those in the organic supply chain are particularly vulnerable if there is opacity in their supply chains. Some studies have considered the role of OCGs in food fraud [117], but this needs further investigation in future studies. Emergent research is exploring ‘organized food crime’ and the vulnerability of food and agricultural sectors to illegal OCG infiltration, but this extends beyond food and farm practice fraud to capture people trafficking, and poor labor practices and money laundering too [117]. To address the interaction between illegal organizations and legitimate businesses, the legal definitions of food crime need to be reframed [117] and there needs to be international consensus on what food fraud is, and legal definitions of food crime. The examples of organic food fraud in this study also show that large scale activities are trans-national, and investigative actions are often required incorporating Europol or Interpol to determine the scale and complexities of the operations. Whilst the fraud diamond is of value [86] to determine organizational vulnerability to food fraud, perpetrators motivations extend much further and, in some cases, outlined the supposed guardians were in themselves involved in the criminal activity.

What lies at the heart of the vulnerability? Reliance on institutional trust across complex supply chains has been highlighted as a recurring theme. The European Commission Action plan for the organic food sector (2021) recognizes this vulnerability and has put forward several actions to safeguard organic food supply chains from fraud [118]. These are to:

- Ensure a robust supervision of control systems in Member States and third countries; increasing cooperation with Member State administrations and third countries;
- Assist Member States in developing and implementing an organic fraud prevention policy using workshops, share lessons learnt and best practices;
- Cooperate with the EU Food Fraud Network and Europol in analyzing the sector to prevent fraud and coordinate investigations; and increase cooperation with competent authorities and law enforcement bodies in third countries;
- Support Member States with guidance on reinforced imports control at border inspection points;
- Promote stronger measures to tackle fraudulent practices through the sanction catalogues;
- Put in place measures to inform consumers and/or to recall from the market products where fraud is identified;
- Develop early warning systems, using artificial intelligence for data mining in EU and Member State databases;
- Develop a database of (organic) certificates of all EU operators, and then relevant third country operators;
- Promote the enrolment of competent authorities and control bodies and the signing of certificates of inspection in TRACES digitally;
- Coordinate regular traceability exercises on organic products in cooperation with Member States, their control bodies and third countries, especially in cases of food fraud suspicion; and
- Assess to what extent the traceability of organic products could benefit from blockchain, digital product passports or other digital technologies.

These actions demonstrate the need to address the range of vulnerabilities already identified in this study to ensure consumer trust in organic foods is maintained and justified.

## 6. Conclusions

Organic foods are credence-based foods and consumer trust is the basis for a positive and dynamic future market developments. The aim of this research has been to explore cases of fraud associated with organic food that are emergent from academic and gray literature searches to identify ways to strengthen future capabilities to counter illicit activities in a globalized food environment. This paper has indicated difficult issues which should be addressed to facilitate consumer trust in food and the integrity of food supply chains, e.g., harmonization of food fraud and food crime definitions at the EU and international level, convergence of private and regulatory approaches to food crime classification, effectiveness, and efficiency of system of sanctions to deter (potential) perpetrators. Case study analysis was used to provide insight into previous organic food incidents and to explore their complex nature. This study provides a framing to consider the potential for food fraud using the Wolf and Hermanson’s four-element fraud diamond model and publically available information on organic food incidents. Furthermore, the paper demonstrates that there is a role for both the public and private sector to come together to develop strong guardianship in organic food supply chains including within the regulatory and enforcement sphere using an effective surveillance system (Table 2). We suggest that the effectiveness of global, regional, or national food surveillance systems can be improved by developing and implementing a combined strategy agreed between government and industry. Public-private partnerships (PPPs) between regulators and food business operators are just one example of such a collaboration. PPPs have great potential to provide added value to the consumer and the public at large [119]. Engaging both public and private players and utilizing data mining and information surveillance techniques can rapidly detect potential instances of food fraud in the organic food supply chain. The data that need to be collated come from a wider variety of sources and need to be independently verified. The involvement of different supply chain actors, including consumers, in a surveillance network could significantly improve information sharing. This would contribute to supply chain and market transparency, and mitigation of food fraud. One example of consumers’ involvement in the flow of information related to foodborne events is the U.S. Department of Agriculture (USDA) Consumer Complaint Monitoring System (CCMS), a database established in 2001 to record, evaluate, and track all consumer food complaints involving meat, poultry, and egg products. This system rapidly organizes and analyzes the incoming complex, multivariate data from consumer complaints which have a wider use for surveillance, outbreak monitoring, and food recalls [120,121]. Development of multi-data food surveillance systems including both public institutions and supply chain actors might result in devising effective prevention measures that are useful to counter the illicit activities.

A limitation of this study approach is the risk of selection bias, and that the search was only undertaken in the English language, so some vital cases could have been lost. Moreover, only publicly available information drawn mainly from the gray literature was considered. Each case was considered in terms of perpetrator motivations (economic, social and behaviorally orientated drivers), the mode of operation (simple or organized), the guardians involved/absent and the business and supply chain level vulnerabilities in order to identify recurrent problems arising in the business of organic food fraud including, inter alia, vulnerability of institutional trust, information asymmetry in the supplier–customer (perpetrator–victim) relationship where opacity influences motivation to commit fraud with organic foods. Several analyzed cases show the lack of effective guardians and the weakness in the mediating role of institutional trust co-owned by regulators, enforcement, private certification and public/private surveillance systems. There were even cases where guardians were involved in the crime itself. These fundamental problems relating to complicity of guardians (organizational and regulatory) erodes public confidence in organic foods and need to be addressed. Further work could be undertaken in the future to contribute to finding an overall solution to the potential for weak guardianship in organic food supply chains and what alternative mechanisms could be adopted to assure consumers that they are getting what they believe they are paying for. Moreover, tailor-made recommendations could be prepared by the European Commission, as they implement their organic supply action plan to increase organic food supply chain integrity; and reduce the likelihood of fraud.

## Figures and Tables

**Table 1:** Motivating factors that influence food fraud in the organic sector (adapted from: [15]).

Economic Drivers	Cultural and Behavioral Drivers
Supply volume and pricing of raw materials	Ethical business culture of the food sector
Valuable components or attributes i.e., degree of economic differential between food product and substitute—the greater the differential the greater the motivation for fraud	History of non-compliance in the food sector
Price asymmetries	Organizational strategy of procurer (own company)
Level of competition in the food sector	Criminal offences associated with customer
Economic health of food sector	Organizational strategy of supplier
Economic health of supplier	Ethical business culture of the supplier
Economic health of procurer (own company)	Criminal offences associated with supplier
Financial strains imposed on supplier by the procurer (own company)	Victimization of supplier
	Corruption level in country of origin of supplier
	Corruption level for country of procurer (own company)
	Criminal offences associated with procurer (own company)
	Ethical business culture of procurer (own company)

**Table 2:** Case studies synthesis matrix: motivations, scope, mode of operation, guardians, and vulnerability.

	Perpetrator Motivations		Scope	Mode of Operation	Guardians		Vulnerability
	Economic	Cultural/Behavioral	National/Global	Simple/OCG	Regulator and Enforcement	Surveillance	
Organic feed incident US, (2011–2017) Conventional product used to dilute organic product or whole consignment sold as organic when conventional.	Economic differential between organic and conventional products. Price asymmetries	Organizational ethical culture History of non-compliance in the food sector	National	Simple	USDA	Retailer Supply chain actors	Weak institutional trust, regulation, enforcement, and surveillance systems
Organic raspberries incident, Chile (2017) Conventional product mislabeled and sold as organic.		Organizational ethical culture. History of non-compliance in the food sector	Global	Evidence suggests simple not an OCG	Chilean Customs Canadian regulators	Supply chain actors	Long and complex supply chains relying on ‘institutionalized’ trust Whistle-blower identified issue so required insider to notify authorities
Organic egg incident UK and Germany (multiple dates) Conventional product mislabeled as organic product.	Economic differential between organic and conventional products. Price asymmetries	Organizational ethical culture History of non-compliance in the food sector	National	Evidence suggests simple not an OCG	German/UK Government and EU regulators	Supply chain actors	Examples not related but again show the vulnerability of institutional trust
Organic pistachios incident, Spain (2019) Conventional product used to dilute organic product or whole consignment intentionally mis-sold as organic.	Economic differential between organic and conventional products. Price asymmetries Laundering money	Ethical culture at the network level Corruption at the organizational and network level	Global	OCG	Spanish Guardia Civil, French Gendarmerie Nationale; Europol	Supply chain actors	Intentional modus operandi to deceive
Puss in Boots, Green War, Vertical Bio incident, Italy, (2007–2011) Highly sophisticated OCG network spanning multiple countries and legal jurisdictions.		Ethical culture at the network level Corruption associated with land title deeds, land lease agreements, creating short term businesses	Global	OCG	Italian Ministry of Agriculture, Central Inspectorate for the protection of food quality and prevention of fraud (ICQRF), but investigated by the Italian Financial Guard	Supply chain actors	Intentional modus operandi to deceive. Guardians (certification bodies) being involved in the fraudulent activity creates a particular vulnerability for consumers

## Data Availability

Data sharing not applicable.
